# Xenomicrobiology: a roadmap for genetic code engineering

**DOI:** 10.1111/1751-7915.12398

**Published:** 2016-08-04

**Authors:** Carlos G. Acevedo‐Rocha, Nediljko Budisa

**Affiliations:** ^1^ Biosyntia ApS2970HørsholmDenmark; ^2^ Novo Nordisk Foundation Center for BiosustainabilityTechnical University of Denmark2970HørsholmDenmark; ^3^ Department of ChemistryTechnical University BerlinMüller‐Breslau‐Str. 10Berlin10623Germany

## Abstract

Biology is an analytical and informational science that is becoming increasingly dependent on chemical synthesis. One example is the high‐throughput and low‐cost synthesis of DNA, which is a foundation for the research field of synthetic biology (SB). The aim of SB is to provide biotechnological solutions to health, energy and environmental issues as well as unsustainable manufacturing processes in the frame of naturally existing chemical building blocks. Xenobiology (XB) goes a step further by implementing non‐natural building blocks in living cells. In this context, genetic code engineering respectively enables the re‐design of genes/genomes and proteins/proteomes with non‐canonical nucleic (XNAs) and amino (ncAAs) acids. Besides studying information flow and evolutionary innovation in living systems, XB allows the development of new‐to‐nature therapeutic proteins/peptides, new biocatalysts for potential applications in synthetic organic chemistry and biocontainment strategies for enhanced biosafety. In this perspective, we provide a brief history and evolution of the genetic code in the context of XB. We then discuss the latest efforts and challenges ahead for engineering the genetic code with focus on substitutions and additions of ncAAs as well as standard amino acid reductions. Finally, we present a roadmap for the directed evolution of artificial microbes for emancipating rare sense codons that could be used to introduce novel building blocks. The development of such xenomicroorganisms endowed with a ‘genetic firewall’ will also allow to study and understand the relation between code evolution and horizontal gene transfer.

## Xenobiology research directions

Biology is an analytical and informational science that is becoming increasingly dependent on chemical synthesis. The high‐throughput and low‐cost synthesis of DNA, for example, is the foundation of the field of synthetic biology (SB). The aim of SB is to provide urgent biotechnological‐based sustainable solutions to problems in the health, energy and environmental sectors (Acevedo‐Rocha, 2016). This sort of biotechnology is performed under the frame of naturally existing chemical building blocks. Compared with the rich methods of synthetic organic chemistry (Walsh *et al*., 2005), however, the diversity of chemistries used by natural organisms is surprisingly narrow; thus, limiting a wide range of useful and chemically diverse biotransformations.

**Figure 1:**
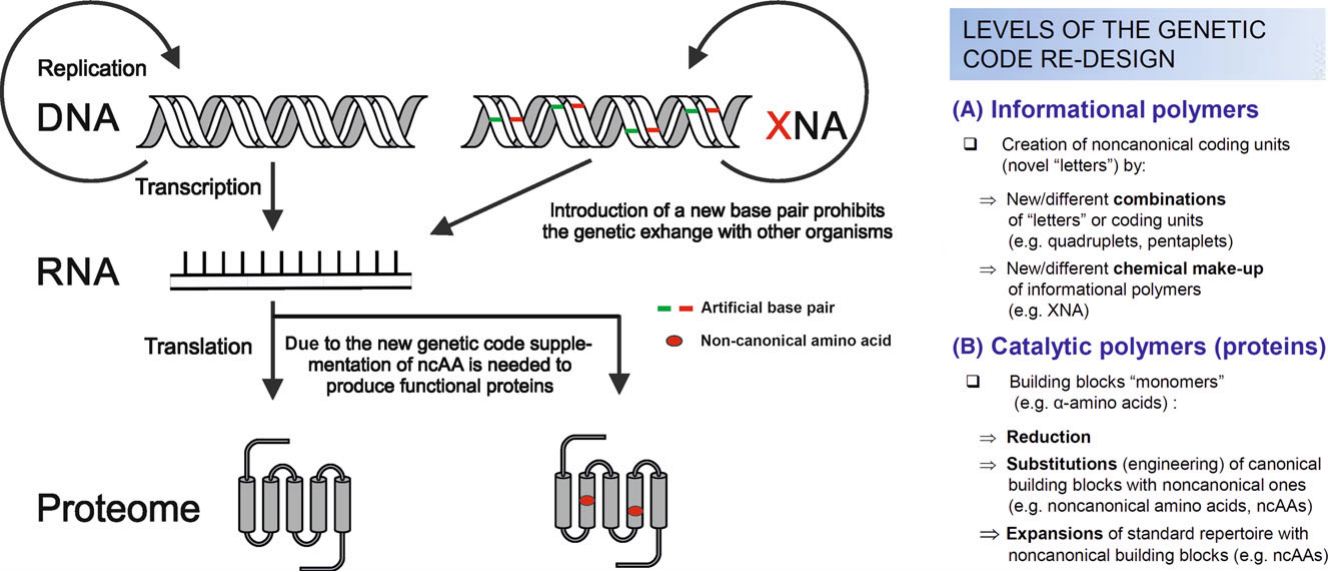
Experimental approaches in xenobiology to re‐design the flow of genetic information. Left: the central dogma of molecular biology is shown. DNA is replicated and transmitted to the descendants or transcribed into RNA. Translation subsequently gives rise to proteins, making up the functional proteome. Recent advances show that the introduction of artificial base pairs as XNA can also replicate and proliferate (Malyshev *et al*., 2014). To date, however, this information cannot be transcribed or translated in vivo. Incorporation of ncAAs into the proteome was shown to be feasible if evolutionary pressure is applied (Hoesl *et al*., 2015). Right: Methods and levels of the genetic code re‐design that enables the partial or full re‐design of informational flow in biology according to Budisa (2014).

To address this issue, the field of xenobiology (XB) aims to endow biological systems with artificial chemistry absent in natural organisms (Schmidt, 2010). XB is a highly diverse area, which two main research directions (Fig. 1). One involves the design and synthesis of alternative nucleic acids (xenonucleic acids, XNAs) based upon new base pairs, sugars and modified backbones (Benner and Sismour, 2005; Pinheiro and Holliger, 2012). The second major area concerns engineering the genetic code of proteins and proteomes with non‐canonical amino acids (ncAAs) (Bacher *et al*., 2004; Budisa, 2004).

Introducing new functional members of XNA into either DNA or RNA and ncAAs into proteins breaks the universality of the genetic code of living organisms, alienating these from other natural life forms (Budisa, 2014). This alienation gives access to possible orthogonal life because both living systems cannot share information with each other, i.e. a ‘genetic firewall’ is built (Acevedo‐Rocha and Budisa, 2011). Furthermore, these synthetic organisms are not able to grow if they are not supplied with synthetic nutrients (trophic containment) (Marliere, 2009). Combination of a genetic firewall with an ‘alien ersatz’ increases the degree of biocontainment (Herdewijn and Marliere, 2009).

Notably, the difference between SB and XB is that the former generates genetically modified organisms (GMOs) based on natural chemical building blocks [e.g. canonical amino acids (cAAs)], whereas the latter aims at creating chemically modified organisms (CMOs) in which the chemical building blocks are artificial (Acevedo‐Rocha, 2016). The likelihood of spreading genetic material from GMOs/CMOs into the environment is still underestimated and not well studied (Schafer *et al*., 2011). Thus, biocontainment of GMOs/CMOs is an active research area for enhancing biosafety in XB (Schmidt and de Lorenzo, 2016), among many other SB‐based research endeavours (Moe‐Behrens *et al*., 2013). The idea of ‘biocontainment’ is of particular interest, as it represents a tool for understanding the relationship between the evolution of the genetic code and horizontal gene transfer. This area is well‐debated in the field of molecular evolution (Gogarten and Townsend, 2005).

In this perspective, we briefly discuss the origin and evolution of the genetic code from the point of view of XB. We then describe the various strategies for engineering the genetic code, including the use of amino acid auxotrophic strains, suppression of stop codons, reassignment of sense codons and reduction of the standard amino acid repertoire. We also highlight the efforts on metabolic, genome and strain engineering for improving efforts for engineering the genetic code. Finally, we discuss the importance and challenges ahead for the directed evolution of xenomicroorganisms towards the understanding of evolutionary innovation and for enhancing biocontainment strategies.

## Origin and features of the genetic code

The genetic code is universal in all three domains of life eukaryotes, bacteria and archaea (Woese *et al*., 1990). It allows the transmission of genetic information stored in DNA from one generation to the next one. Transcription of protein‐encoding genes into RNA enables the translation machinery to convert this information into proteins, which are the executors of the genetic information. A reliable and accurate translation of the linear RNA‐sequence into a functional protein is ensured by tRNAs. These adaptor molecules read the mRNA in a three‐base letter sequence code, i.e. in triplets (Crick *et al*., 1961).

**Figure 2:**
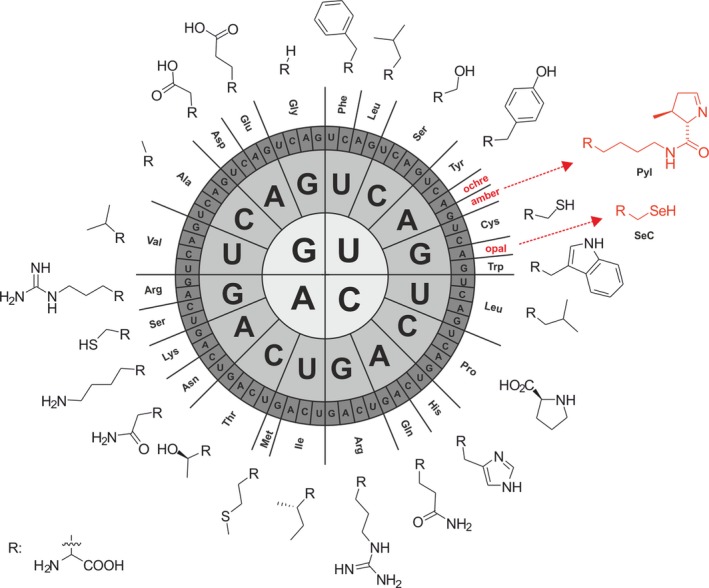
The genetic code structure in the RNA format in a radial representation. Chart from inside to outside: a triplet of mRNA (5′→3′) is assigned to one of the 20 canonical amino acids or a stop codon. The natural expansion of the genetic code of selenocysteine (SeC) at opal and pyrrolysine (Pyl) at the amber and opal stop codons is depicted (Courtesy provided by Dr Stefan Oehm) (Oehm, 2016).

The chemistry of the universal translational apparatus is highly standardized: DNA consists of four different bases (A, T, G and C) and there are 4^3^ = 64 possible triplet combinations for encoding 20 cAAs. An important feature of the genetic code is its codon degeneracy, i.e., the genetic code is redundant because several triplets can code the same amino acid (Lagerkvist, 1978). Briefly, there are 61 sense codons coding for amino acids and 3 stop codon signals (UAA: ochre, UGA: opal and UAG: amber) that are responsible for terminating protein biosynthesis at the ribosome (Fig. 2). The assignment of one triplet to one amino acid is not random: the degeneracy of the genetic code enables an organism to choose between abundant and rare codons. The number of triplets is often directly proportional to the abundance of the amino acid in the proteome. For example, the amino acid tryptophan, which is relatively rare (present in approx. 1.5% in the proteome of *Escherichia coli*), is only encoded by the single UGG codon, whereas arginine (around 5.5% distribution in the *E. coli* proteome) is decoded by six triplets (Lajoie *et al*., 2013).

In *E. coli*, out of the 61 sense codons, two are unique (methionine and tryptophan) while the rest are degenerate, with amino acids being encoded by as little as two and as much as six triplets (Fig. 2). In the latter case, several of the codons are rare (e.g., arginine) and allow an organism to control the expression rate of proteins, thus providing a selective advantage when adapting to a changing environment (Subramaniam *et al*., 2013a). For example, protein translation of abundant and rare codons seems to be equally fast when *E. coli* grows in medium rich of amino acids. In nutrient‐deficient media, by contrast, translation velocity of rare codons is substantially decreased, suggesting that the intracellular amino acid concentration is important for efficient reading of rare codons and not the isoacceptor tRNA abundance (Li *et al*., 2012; Subramaniam *et al*., 2013b; Ling *et al*., 2015).

An unresolved question is why the 20 cAAs were selected by evolution even though several other non‐proteinogenic amino acids were available in the ‘primordial soup’ (Kvenvolden *et al*., 1971) including norvaline and norleucine? Special cAAs can be added to the standard amino acid repertoire. For instance, many organisms use the 21st (selenocysteine, SeCys, or Sec) and 22nd (pyrrolysine, Pyl) amino acids (Fig. 2) for adaptive purposes (Chambers *et al*., 1986; Srinivasan *et al*., 2002).

The evolution of the genetic code is a historical process and we will most probably always remain agnostic about its origin and sequence of events that lead to the current codon–amino acid associations. Upon the elucidation of the genetic code in 1964 (Nirenberg and Leder, 1964), Crick postulated a ‘frozen accident theory’ which assumes that the association of particular amino acids to their codons was accidental until the point where any further member expansion would be lethal to cells due to functional protein destabilization (Crick, 1968). This concept has been questioned for many times during the last decades; the plasticity and evolvability of the standard genetic code is postulated to be plausible, as supported by the finding of distinct alternative genetic codes in prokaryotes (Oba *et al*., 1991), eukaryotic nuclear genomes (Sugita and Nakase, 1999) and mitochondrial genomes (Inagaki *et al*., 1998). Additional widely accepted concepts about the origin of the genetic code include stereochemical (Pelc, 1965), adaptive (Woese, 1965) and co‐evolution (Wong, 1975, 2005) theory.

**Figure 3:**
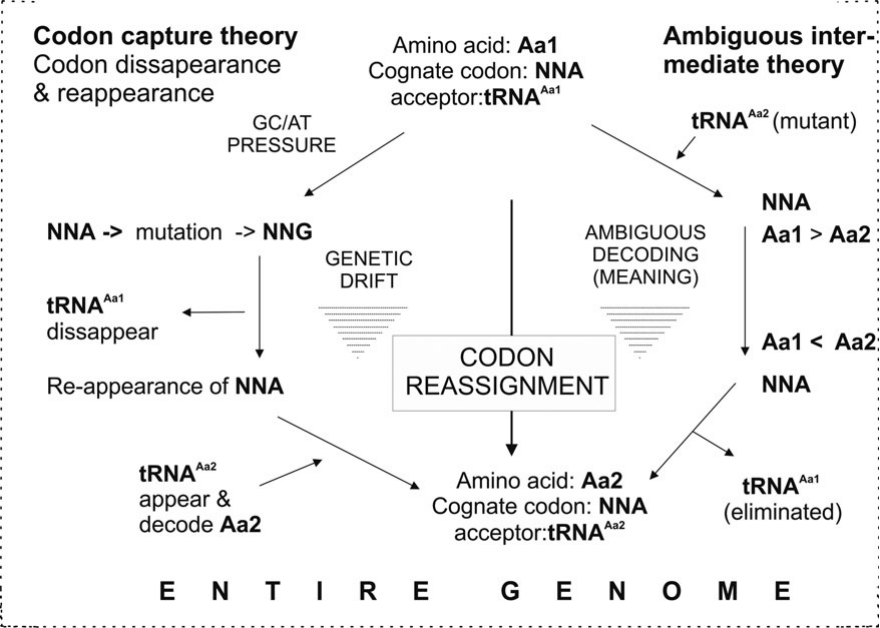
The codon capture and ambiguous intermediate theory for codon reassignment in the historical development of the genetic code. While in codon capture (left) the endogenous tRNA disappears due to the removal of its designated codon, in ambiguous intermediate theory (right) the exogenous tRNA is in direct competition with the cognate one. In the end, both theories end up in a reassigned codon. Figure modified according Santos *et al*. (2004) and Budisa (2006a).

Before it was ‘frozen’, the early genetic code underwent evolution and expansion, which is encompassed by two main theories about codon reassignment. Figure 3 summarizes them and explains possible evolutionary mechanisms for codon reassignment to other amino acids: codon capture (Osawa *et al*., 1992) and ambiguous intermediate (Schultz and Yarus, 1994) theory. The codon capture theory postulates that genomic composition changes due to GC pressure could impose alternative codon usage mainly due to changes at the third base position of a codon. Owing to this pressure, a potential codon can disappear, making a particular cognate tRNA obsolete. Reappearance of the codon due to random drift would lead to its recognition by another tRNA (that could also charge another amino acid) leading to codon reassignment, i.e., change the identity of the codon. Conversely, the ambiguous intermediate theory postulates an appearance of mutant tRNA in competition with the cognate tRNA. In the course of selective pressure, the latter might lose the race against the mutant tRNA, hence giving rise to another amino acid meaning [see Fig. 3 and (Budisa, 2006a)].

## Code and metabolic engineering of auxotrophic strains

**Figure 4:**
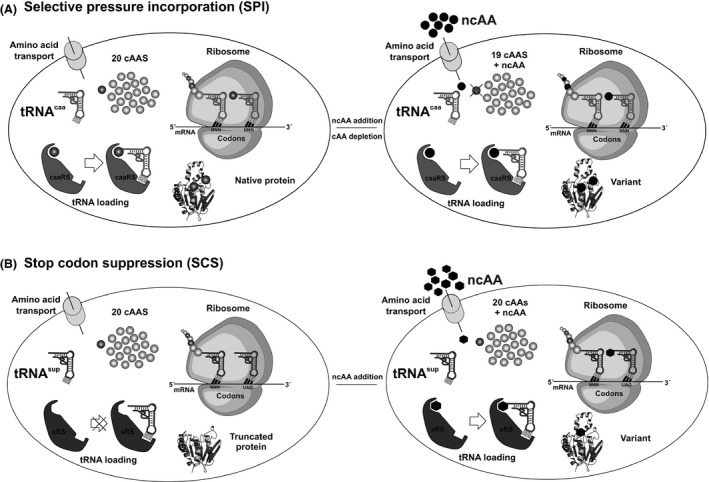
Flow chart presentations of two basic in vivo approaches for incorporating non‐canonical amino acids into proteins. (A) Auxotrophy‐based selective pressure incorporation (SPI) method exploits the endogenous translational system to load an isostructural analogue onto a canonical tRNA leading to residue‐specific incorporation, i.e. cAA→ncAA substitution. (B) Stop codon suppression (SCS) usually endows the host with a new orthogonal aaRS:tRNA pair in charge of introducing a given ncAA or more at one or more stop codons. For more details see Hoesl and Budisa (2012).

Genetic code engineering aims to use ncAAs as building blocks in proteins. It exploits the flexibility of the components involved in protein translation mainly aminoacyl‐tRNA synthetases (aaRSs) and tRNAs (Fig. 4). While many aaRSs have evolved mechanisms to differentiate between naturally abundant and structurally related amino acids (especially metabolic intermediates) (Schmidt and Schimmel, 1994), most ncAA that can be incorporated with this approach do not occur in natural environments. In the laboratory, however, a codon can be read ambiguously by mischarging tRNAs (i.e. a cognate tRNA can be charged with two different yet similar substrates). For example, methionyl‐tRNA synthetase charges its cognate tRNA^Met^_CAT_ with a variety of methionine (Met) analogues, homologues and surrogates into heterologous‐expressed proteins (Wiltschi *et al*., 2009). The best know example is selenomethionine (SeMet), which can almost quantitatively replace Met in proteins and proteomes in suitable auxotrophic strains (Cowie and Cohen, 1957; Budisa *et al*., 1995). We and others have shown many examples of potential applications of such an approach for Met analogues and beyond during the last two decades (Mohammadi *et al*., 2001; Link and Tirrell, 2005; Acevedo‐Rocha *et al*., 2013; Bohlke and Budisa, 2014; Piotrowski *et al*., 2015). In another example, the incorporation of norleucine (Nle) into a lipase allow the engineering of a highly active ‘cold‐wash’ enzyme (Hoesl *et al*., 2011).

Till date, more than 50 ncAAs (mostly Met, Trp, Tyr, Phe, Pro, Arg, Lys analogues) (Budisa, 2006b) (Fig. 4A) have been incorporated into proteins using this approach. Unfortunately, most of these experiments are limited to individual xeno‐proteins overexpressed from auxotrophic strains. Although this is an important research area that can give rise to potential applications in biocatalysis, it is important to consider the costs of supplying expensive ncAAs into a big fermenter. For this reason, genetic code engineering can be combined with metabolic engineering. For example, we implemented a biosynthetic route in for the biosynthesis of the ncAA azido homoalanine (Aha) by expressing in trans O‐acetylhomoserine sulfhydrylase (cgOAHSS) from *Corynebacterium glutamicum* in the Met‐auxotroph *E. coli* strain B834 while supplying the strain with *O*‐acetyl‐homoserine and inorganic sodium azide (NaN_3_) as precursors (Ma *et al*., 2014). This engineering effort resulted in an approach for a more economical production of Aha‐labelled proteins, which can be selectively modified via ‘click chemistry’ with different molecules for protein immobilization or labelling (De Simone *et al*., 2016). Likewise, the group of Wiltschi recently afforded a strain able to produce Nle‐labelled proteins in sufficient economically amounts (Anderhuber *et al*., 2016). This kind of experiments will become more important if applications of ncAAs in biocatalysis are likely to succeed. Ideally, the precursors of the ncAA should be biosynthesized starting from a cheap carbon source such as glucose. We envision more synergies between metabolic and genetic code engineering in the years to come.

## Code engineering by stop codon suppression

In contrast to the ambiguous decoding of sense codons by ncAAs as explained above, the meaning of a stop codon can be suppressed by introducing engineered orthogonal pairs (Fig. 4B). The most common pairs are the *Methanocaldococcus jannaschii* TyrRS:tRNA^Tyr^_CUA_‐pair, and PylRS:tRNA^Pyl^_CUA_‐pairs from *Methanosarcina barkeri* and *Methanosarcina mazei*. This stop codon suppression (SCS) approach allows the site‐specific incorporation of ncAAs into a target protein, which is usually overexpressed (Hoesl and Budisa, 2012). Because alteration of the protein structure is minimal, it is possible to study protein function with an unprecedented level of accuracy. For example, it is possible to study protein conformation by Förster resonance energy transfer, follow intracellular protein localization (using fluorescent ncAAs as probes) or determine protein–protein interactions by using cross‐linking ncAAs (Lang *et al*., 2015).

The SCS methodologies have matured in the last years, offering researchers with a plethora of ncAAs to incorporate site‐specifically (Wright *et al*., 2016). However, the SCS is usually limited because the release factors (RFs), which are responsible for terminating protein biosynthesis at the ribosome, compete with the tRNAs, resulting in a mixture of labelled and truncated protein. Although the efficiency of ncAA incorporation can be improved with RF‐free engineered strains (Amiram *et al*., 2015), additional ‘context effect’ issues persist, which is still the most probably cause of obtaining low protein yields. Recently, Ignatova and others provided a more precise experimental description of these phenomena. The insertion of a stop codon in a particular sequence can cause large deviations from natural mRNA folding energy. Furthermore, it can affect the binding site interaction strength within the ribosome (see e.g. Gorochowski *et al*., 2015). In the years to come, we expect to obtain better in silico models that could predict what the most suitable sites for inserting stop codons are. Hence, promoting synergies between computational chemistry and code engineering is an important research endeavour.

## Code engineering by sense codon reassignment

Codon reassignment mainly includes the removal of the endogenous translation components like tRNA and aaRS, synonymous codon swaps throughout the chromosome or essential genes (Lajoie *et al*., 2015). For example, the groups of Isaacs (Rovner *et al*., 2015) and Church (Mandell *et al*., 2015) engineered the genetic code of *E. coli* C321.ΔA (which lacks amber stop codons) by re‐introducing amber stop codons into essential genes (devising a sort of experimental ‘codon capture’ approach). Because the UAG codon lacks any meaning in that distinct strain, these otherwise lethal stop codons have to be rescued with *M. jannaschii* TyrRS:tRNA^Tyr^_CUA_ for *p*‐azido‐l‐phenylalanine (pAzF) or biphenyl‐l‐alanine (bipA) (Xie *et al*., 2007) respectively. As the UAG codon has no meaning in strain C321.ΔA, both studies converted (‘captured’) this codon into a sense one by supplying orthogonal pairs. Although both experiments differ in their strategy and their execution, they yield the same result: a GMO that cannot grow without supplying the ncAA, therefore becoming trophically biocontained. Those synthetic auxotrophic strains show less than 6 × 10^−12^ escape mutants per colony forming unit in various media. Most other techniques for biocontainment purposes yield higher escape frequencies and suffer from the possibility of cross‐feeding of metabolites produced from other organisms (Schmidt and de Lorenzo, 2016). Both ncAAs used (pAzF and bipA) are not available in natural environments. However, the synthetic auxotrophy only relies on the orthogonal translation system. This system might be stable under laboratory conditions, but there are many places where the system could lose its orthogonality, e.g., mutating TyrRS to accept any other cAA, introduction of a natural amber suppressors (*sup*E, *sup*D, *sup*F, *sup*Z, etc.) or simply reverting the amber sites back to their original meaning. Thus, long‐term evolution experiments of strain C321.ΔA in more diverse and rich media are needed to ensure that it can be biocontained without reversing its genotype.

While ambiguous decoding and SCS approaches are always in competition with the original codon meaning and function of the corresponding amino acid, sense codon reassignment aims to completely remove the pre‐existent functional information of a certain codon. For example, isoleucine can be coded by codon AUA, but there is no tRNA^Ile^_UAU_ gene in *E. coli* because the third base of the tRNA^Met^_UAC_ is modified by tRNA^Ile^‐lysidine synthetase (TilS) to lysidine, yielding tRNA^Ile^_LAU_ that is read by wobbling at the third base position (Bohlke and Budisa, 2014). We generated a *tilS* knockout strain (this mutation is lethal to the cell leaving more than 5000 codons unassigned) by using a rescue plasmid expressing the *Mycoplasma mobile* IleRS:tRNA^Ile^_UAU_ pair to sustain cell viability. The rescue plasmid can be easily removed in *E. coli* ΔtilS and can be exploited to apply evolutionary pressure to reassign the Ile codon AUA to another cAA (Doring and Marliere, 1998) or even ncAA.

Another strategy was reported by Sakamoto and co‐workers (Mukai *et al*., 2015) who reassigned the rare AGG codon of arginine (Arg) to l‐homoarginine (hArg) and l‐N6‐(1‐iminoethyl)lysine (l‐NIL) by using PylRS:tRNA^Pyl^_CUA_‐pair. Cells can survive the deletion of Arg rare codons (*arg*W and *arg*U) only if the evolved PylRS‐system is co‐expressed and AGG positions in most essential genes are exchanged by synonymous codons (to avoid detrimental effects of AGG‐reading as hArg or l‐NIL). Although analytical data were not presented (e.g. MS/MS experiments in essential genes to verify Arg‐replacement), the exchange of Arg at remaining AGG positions most probably took place given that both ncAAs used (hArg, l‐NIL) are similar to the cAA Arg (‘similar replaces similar’) (Moroder and Budisa, 2010).

## Code engineering via reduction of the standard amino acid repertoire

Widely accepted co‐evolution theory about the origin of the genetic code postulates its evolution from a simplified set of amino acid and extension to recent form with the expansion of cellular metabolism (Wong, 1981). Therefore, an experimental model with the reduced genetic code could provide some insight into evolutionary trajectories of amino acids. This was accomplished by the construction of a functional enzyme using a nine‐amino acid alphabet (Walter *et al*., 2005). Namely, Hilvert and colleagues demonstrated that *E. coli* lacking the aroQ gene for chorismate mutase is unable to grow in media where all sources for tyrosine (Tyr) and phenylalanine (Phe) are withdrawn. This synthetic auxotrophy can be exploited to screen a library of artificial chorismate mutase analogues, in which aroQ is only composed from a nine‐amino acid alphabet. It was found that even this reduced set of amino acids can lead to a functional, metabolically competent and near to wild‐type growing strain. However, the reduction led to a restricted diversity ending up with a destabilization of overall protein structure. These results indicate the need of more amino acids for robust proteins and explain the evolutionary force towards the increased diversity. A more general approach towards this issue was reported by Marlière and co‐workers: they showed the possibility of removing Trp from the genetic code (Pezo *et al*., 2013). Taking advantage of the conserved and essential histidine (His) residue in the active site of yeast transketolase gene *tktY*, this position was mutated to the Trp‐codon UGG in *trans* in a suitably configured *E. coli* MG1655 ΔtktAB strain. Supplying this strain with the missense tRNA_His_^CCA^, the UGG codon in the essential TktY is read as a His leading to a functional gene, which is the driving force to keep the missense tRNA. Subsequently, the system was proliferated in a continuous pulse feeding regime, yielding 30 times higher mis‐incorporation of His at Trp positions in proteins. This experiment suggests the possibility of engineering the genetic code applying evolutionary pressure using a reduced amino acid alphabet. This is an important research area whose potentials have not yet been extensively studied (Oehm, 2016).

## Code engineering via experimental evolution

Over more than three billion years, the standard genetic standard code has not undergone any significant changes besides some local alterations (Knight *et al*., 2001). Therefore, an utmost goal of XB is to introduce new biochemical building blocks into an organism at both the genomic and proteomic level. One option is by performing experimental evolution, which deals with the long‐term cultivation of organisms under controlled conditions. Doubtless, adaptive evolution experiments with suitably designed metabolic prototypes could accelerate the propagation, manipulation and analysis of organisms in a controlled environment. Furthermore, such evolutionary adaptation is the most expedient route to generate artificial biocontained microbes. Because microbes have fast generation times and large population sizes in a reasonable and manageable dimension of cultivation space, they are ideal candidates for such experimental setups. Additionally, populations can be easily frozen for later analysis (‘fossils’). Adaptive evolution experiments have been designed to cultivate *E. coli* to adapt on glucose, glycerol and lactate minimal medium, high temperature irradiation as well as high salt or ethanol concentration (Blount *et al*., 2012; Wiser *et al*., 2013).

Already in the late 1950s, Cowie and Cohen (1957) demonstrated that Met auxotrophic *E. coli* grow in Met‐free medium when SeMet is supplied. This proteome wide exchange of Met by producing functionally essential enzymes for cell proliferation illustrates how the amino acid composition of a proteome can be influenced by external pressure. Another report from 1963 by Rennert and Anker claims that *E. coli* cells able to use 5′,5′,5′‐trifluoroleucine (TFL) as an analogue for leucine (Leu) can be isolated from a suitably designed cultivation experiment (Rennert and Anker, 1963). Wong (1983) was able to adapt a Trp deficient *Bacillus subtilis* with some few single‐cell transfers to grow on 4‐fluoro‐l‐tryptophan (4F‐Trp) as Trp source. Using the mutagenic agent it was possible to obtain a derivative exhibiting preference towards the ncAA. Nevertheless, those old experiments were not accompanied by precise analytic measurements to quantify the extent of ncAA incorporation (contaminations of cAA from media preparation cannot be excluded – most likely, those sources of cAA were elemental for the observed growth). Moreover, those experiments were conducted in ‘rich’ medium in which standard amino acids, vitamins and nucleobases were supplemented. Therefore, only a small portion of the cellular machinery had to adapt to the incorporation of the ncAA. Bacher and Ellington (2001) adapted the *E. coli* strain C600 ΔtrpE towards 4F‐Trp by serially transferring it to minimal medium while gradually increasing the 4F‐Trp‐to‐Trp ratio. Unfortunately, the commercial source of 4F‐Trp was contaminated with 0.03% Trp (the authors were not able to withdraw all cAA‐source from the media). Yet they found a strain tolerating high levels of 4F‐Trp to a level at which it was toxic for the parental strain. Due to the lack of next generation sequencing, only a few relevant genes were sequenced but these were unchanged (*tna*A, *trp*R, *mtr*,* trp*T, *bla*,* gap*A), while others like *trp*S, *aro*P and *tyr*R exhibited single nucleotide polymorphisms. Expression of *trp*S and *aro*P mutant proteins in *trans* in the ancestral strain led to a growth advantage in the presence of 4F‐Trp. However, those mutations are not sufficient to let the C600 strain incorporate the ncAA at high levels (deep analytics were not performed). Nevertheless, this experiment improved our understanding of serial evolution experiments to change the chemical composition of the proteome.

Based on the experiences gathered on earlier long‐term *E. coli* cultivation experiments by the groups of Lenski, Ellington, Bacher and Wong, we also set out to adapt this bacterium towards an ncAA. In particular, our group completely substituted Trp by the analogue Thienopyrrole‐alanine ([3,2]Tpa) in a Trp auxotrophic strain (Hoesl *et al*., 2015). To avoid contamination of Trp traces in commercial ncAA preparations, the strains were engineered to produce the surrogate by using the enzyme tryptophan synthase (TrpBA), which condensates the indole analogue [3,2]Thienopyrrole with serine yielding [3,2]Tpa (Budisa *et al*., 2001). Rigorous and high‐precision analytics confirmed the almost completely quantitative replacement of Trp by the surrogate at 20 899 sense codons in *E. coli* W3110 genome. Hence, the engineered strain can be defined as ‘trophically reassigned’ (i.e., the meaning of a codon is redefined throughout the whole proteome). However, supplementing these cells with Trp reverses them to ‘natural’ ones given that they still favour the incorporation of the canonical building block. To achieve a Trp‐independent reassignment (i.e. ‘real’ codon reassignment) at all UGG codons across *E. coli*'s genome – an experimental strategy for biocontainment still needs to be developed and executed (Acevedo‐Rocha and Schulze‐Makuch, 2015).

## Roadmap to equip xenomicroorganisms with a ‘genetic firewall’

In the previous sections, we highlighted various approaches for genetic code engineering. The most popular one is perhaps the genome‐wide replacements of stop or rare sense codons with synonymous alternatives in order to design genomes with radically altered genetic codes, which can be used for biocontainment purposes. However, owing to the complexity of experimental genome re‐design, it is difficult to avoid changes that impair cellular fitness, which creates fragile strains. Among other features, rare codons fine tune translation to facilitate the biogenesis of the encoded protein and their synonymous replacements can lethally impact mRNA secondary structure or ribosome binding sites which is expected to result in dramatically decreased fitness during the genome assembly process (Plotkin and Kudla, 2011). Furthermore, widely used orthogonal pairs are not as active and accurate as natural aaRSs (Nehring *et al*., 2012). An alternative strategy for experimental genetic code evolution relies on the global substitution of cAAs with ncAAs (or an addition of ncAAs) assisted with simple metabolic optimization following an ‘ambiguous intermediate’ engineering concept. In other words, we propose a novel strategy that relies on liberation of rare sense codons of the genetic code (i.e. ‘codon emancipation’) from their natural decoding functions (Bohlke and Budisa, 2014). This approach consists of long‐term cultivation of bacterial strains coupled with the design of orthogonal pairs for sense codon decoding. In particular, directed evolution of bacteria should be designed to enforce ambiguous decoding of target codons using genetic selection. In this system, viable mutants with improved fitness towards missense suppression can be selected from large bacterial populations that can be automatically cultivated in suitably designed turbidostat devices. Once ‘emancipation’ is performed, full codon reassignment can be achieved with suitably designed orthogonal pairs. Codon emancipation will likely induce compensatory adaptive mutations that will yield robust descendants tolerant to disruptive amino acid substitutions in response to codons targeted for reassignment. We envision this strategy as a promising experimental road to achieve sense codon reassignment – the ultimate prerequisite to achieve stable ‘biocontainment’ as an emergent feature of xenomicroorganisms equipped with a ‘genetic firewall’.

## Conclusions

In summary, genetic code engineering with ncAA by using amino acid auxotrophic strains, SCS and sense codon reassignment has provided invaluable tools to study accurately protein function as well as many possible applications in biocatalysis. Nevertheless, to fully realize the power of synthetic organic chemistry in biological systems, we envision synergies with metabolic, genome and strain engineering in the next years to come. In particular, we believe that the experimental evolution of strains with ncAAs will allow the development of ‘genetic firewall’ that can be used for enhanced biocontainment and for studying horizontal gene transfer. Additionally, these efforts could allow the production of new‐to‐nature therapeutic proteins and diversification of difficult‐to‐synthesize antimicrobial compounds for fighting against ‘super’ pathogens (McGann *et al*., 2016).

Yet the most fascinating aspect of XB is perhaps to understand the genotype–phenotype changes that lead to artificial evolutionary innovation. To what extent is innovation possible? What emergent properties are going to appear? Will these help us to re‐examine the origin of the genetic code and life itself? During evolution, the choice of the basic building blocks of life was dictated by (i) the need for specific biological functions; (ii) the abundance of elements and precursors in past habitats on earth and (iii) the nature of existing solvent(s) and available energy sources in the prebiotic environment (Budisa, 2014). Thus far, there are no detailed studies on proteomics and metabolomics of engineered xenomicrobes, let alone systems biology models that could integrate the knowledge from such efforts. In 2020, we expect to have such kind of analysis in order to have a clearer picture of the possibilities that artificial evolution can offer. We believe that expanding the repertoire of the genetic code beyond the canonical 20 (+2) amino acids will not only change the chemical makeup of life, but also allow to re‐examine our understanding and current concepts about the origin of the genetic code and life itself.

## Conflict of interest

None declared.
